# Electronic Couplings and Electrostatic Interactions Behind the Light Absorption of Retinal Proteins

**DOI:** 10.3389/fmolb.2021.752700

**Published:** 2021-09-15

**Authors:** Kazuhiro J. Fujimoto

**Affiliations:** ^1^ Institute of Transformative Bio-Molecules (WPI-ITbM), Nagoya University, Nagoya, Japan; ^2^ Department of Chemistry, Graduate School of Science, Nagoya University, Nagoya, Japan

**Keywords:** color tuning, excitation-energy transfer, electronic coupling, electrostatic interaction, quantum chemistry

## Abstract

The photo-functional chromophore retinal exhibits a wide variety of optical absorption properties depending on its intermolecular interactions with surrounding proteins and other chromophores. By utilizing these properties, microbial and animal rhodopsins express biological functions such as ion-transport and signal transduction. In this review, we present the molecular mechanisms underlying light absorption in rhodopsins, as revealed by quantum chemical calculations. Here, symmetry-adapted cluster-configuration interaction (SAC-CI), combined quantum mechanical and molecular mechanical (QM/MM), and transition-density-fragment interaction (TDFI) methods are used to describe the electronic structure of the retinal, the surrounding protein environment, and the electronic coupling between chromophores, respectively. These computational approaches provide successful reproductions of experimentally observed absorption and circular dichroism (CD) spectra, as well as insights into the mechanisms of unique optical properties in terms of chromophore-protein electrostatic interactions and chromophore-chromophore electronic couplings. On the basis of the molecular mechanisms revealed in these studies, we also discuss strategies for artificial design of the optical absorption properties of rhodopsins.

## Introduction

The photo-functional chromophore retinal can significantly change its optical absorption properties by interacting with surrounding proteins (Wald, 1968; Callender and Honig, 1977; Kochendoerfer et al., 1999; Mathies et al., 2000) and other chromophores (Lanyi and Balashov, 2008; Balashov et al., 2010; Anashkin et al., 2018; Misra et al., 2019). These changes in the optical properties of retinal play an important role in the expression of biological functions such as ion transport and signal transduction in rhodopsin (Birge, 1990; Kandori et al., 2001), which is found in a wide range of organisms from microbes to animals, including humans (Shichida and Imai, 1998; Hampp, 2000; Ebrey and Koutalos, 2001). To clarify the molecular mechanisms underlying these photobiological phenomena from the perspective of theoretical calculations, three points need to be taken into account. The first is to accurately describe the electronic structure of the retinal chromophore in the excited state, the second is to efficiently incorporate the effect of the surrounding protein environment of the retinal chromophore, and the third is to quantitatively analyze the interaction between chromophores in the excited state. The symmetry-adapted cluster-configuration interaction (SAC-CI) (Nakatsuji, 1978; Nakatsuji, 1979a; Nakatsuji, 1979b), combined quantum mechanical and molecular mechanical (QM/MM) (Warshel and Levitt, 1976; Senn and Thiel, 2009; Brunk and Rothlisberger, 2015), and transition-density-fragment interaction (TDFI) (Fujimoto and Hayashi, 2009; Fujimoto, 2010; Fujimoto, 2012) methods are effective tools for considering each of the above three points.

The SAC-CI method (Nakatsuji, 1978; Nakatsuji, 1979a; Nakatsuji, 1979b) is an electronic structure theory (a method of quantum chemistry) proposed by Nakatsuji. This method has the advantage of accurately describing the molecular ground and excited states, and has been applied to numerous molecules. In particular, many spectroscopic studies have been carried out using the SAC-CI method, and the effectiveness of this method has been demonstrated by successfully attributing spectra that were difficult to understand experimentally. It should be emphasized that the SAC-CI method satisfies the requirements of orthogonality and Hamiltonian orthogonality between the ground and excited states for an accurate wave function. Another feature of the SAC-CI program is the use of an efficient computational technique called the perturbation selection method (Nakatsuji, 1983). This has realized a significant reduction in computational cost. The SAC-CI method is implemented in the Gaussian program package (Frisch et al., 2003) and is widely used in chemistry and physics research involving various electronic states.

The QM/MM method (Warshel and Levitt, 1976; Senn and Thiel, 2009; Brunk and Rothlisberger, 2015) is a hybrid approach of quantum chemistry and molecular mechanics, and has been established as an effective tool for describing protein environments. Warshel and Levitt, who proposed the QM/MM method, along with Karplus, won the 2013 Nobel Prize in Chemistry for “the development of multiscale models for complex chemical systems”. Large-scale quantum chemical calculations have been frequently performed on whole proteins using the divide and conquer (DC) method (Yang, 1991; Yang and Lee, 1995) proposed by Yang and the fragment molecular orbital (FMO) method (Kitaura et al., 1999; Nakano et al., 2002) proposed by Kitaura *et al.* However, most of these are single point calculations. Although computational techniques for geometry optimization using these methods have been developed (Zhao and Yang, 1995; Fedorov et al., 2007), their application to whole proteins is not practical due to the huge computational cost. On the other hand, the QM/MM method incorporates the interactions that are intrinsically important in the protein environment into the quantum chemical calculation (i.e., the electrostatic potential from the protein is included in the Fock operator), and treats the other interactions classically. Such an efficient computation by the QM/MM method enables us to perform geometry optimization of proteins. The QM/MM optimization using *ab initio* method (Hayashi and Ohmine, 2000; Hayashi et al., 2001) as well as the semiempirical method (Ren et al., 2001; Bondar et al., 2004) for the QM part was successful for retinal proteins. The QM/MM method is also applicable to excited state calculations, and the excitation energies (i.e., the energy difference between the ground state and the excited state, corresponding to the absorption energy) presented below are obtained with the SAC-CI method for the QM part.

The TDFI method was developed by the author to describe electronic coupling, which is an intermediate physical quantity to explain the intermolecular interaction between different electronic states. The dipole-dipole (DD) approximation (Förster, 1948), a conventional method for electronic coupling calculations, has a simple and intuitive form using intermolecular orientation and intermolecular distance based on transition dipoles, while the DD method has a limitation of application arising from the assumption that the intermolecular distance between donor and acceptor is larger than their molecular sizes (Speiser, 1996). Many computational methods for electronic coupling have been developed to overcome the problem of the DD method (Chang, 1977; Krueger et al., 1998; Tretiak et al., 2000; Hsu et al., 2001; Iozzi et al., 2004; Wong et al., 2004; Madjet et al., 2006; Neugebauer, 2007; Fink et al., 2008; Fückel et al., 2008; Vura-Weis et al., 2010; Kawatsu et al., 2011; Voityuk, 2013; Fujimoto, 2014; Błasiak et al., 2015). The transition density cube (TDC) method (Krueger et al., 1998) developed by Krueger et al. is a pioneering approach for calculating electronic couplings using the transition densities of molecular fragments. Similar to the TDC method, the TDFI method also uses the transition densities of molecular fragments, but with technical improvements such as the use of atomic orbital (AO) two-electron integrals for spatial integration (Fujimoto and Hayashi, 2009; Fujimoto, 2010) and the self-consistent incorporation of interactions between molecular fragments (Fujimoto and Yang, 2008; Fujimoto, 2010). As a result, the TDFI method achieves highly accurate electronic coupling calculations even for systems with small intermolecular distances (Fujimoto, 2010; Fujimoto, 2012). In addition, while the DD and TDC methods can only evaluate the Coulomb interaction in the electronic coupling, the TDFI method can evaluate not only the Coulomb interaction but also the exchange interaction and the higher-order interactions using perturbation expansion (Fujimoto, 2012; Fujimoto and Kitamura, 2013; Fujimoto, 2015). Such an estimate of each component in electronic coupling is useful for analyzing molecular mechanisms.

In this review, we present three topics on the light absorption properties of retinal proteins revealed by the application of the SAC-CI, QM/MM, and TDFI methods (Fujimoto et al., 2008; Fujimoto et al., 2009; Fujimoto and Hayashi, 2009; Fujimoto and Balashov, 2017; Fujimoto and Inoue, 2020): first, the color tuning mechanism of human cone pigments responsible for color vision (Wang et al., 1993; Asenjo et al., 1994; Lin et al., 1998; Fasick et al., 1999; Kandori et al., 2001); second, the excitation energy transfer (EET) that occurs in xanthorhodopsin (XR) (Balashov et al., 2005; Lanyi and Balashov, 2008); and third, the circular dichroism (CD) spectra of XR (Balashov et al., 2006; Smolensky Koganov et al., 2015) and *Krokinobacter eikastus* rhodopsin 2 (KR2) (Inoue et al., 2013; Gushchin et al., 2015; Kato et al., 2015; Shibata et al., 2018). Here, the term color tuning refers to the change in absorption wavelength of a molecule depending on the protein environment (Shichida and Imai, 1998; Coto et al., 2006), and excitation energy transfer (EET) (Scholes, 2003; May and Kühn, 2011) refers to the phenomenon of simultaneous deexcitation of the donor molecule and excitation of the acceptor molecule. All of these topics are related to light absorption by retinal proteins, but note that the major physical factors are different in each system. In the case of human cone visual pigments, electrostatic interactions between retinal and surrounding proteins play a central role, while in the case of XR and KR2, electronic couplings between chromophores such as retinal-carotenoid and retinal-retinal contribute significantly. Quantitative analysis of electrostatic interactions and electronic couplings is indispensable for sufficiently understanding these molecular mechanisms.

## Color Tuning Mechanism of Human Cone Visual Pigments

The human retina contains three types of cone photoreceptors that control color vision: human red (HR), green (HG), and blue (HB) cone pigments. HR, HG, and HB are all retinal proteins, and the retinal chromophores in the proteins are directly involved in the absorption of light. Thus, the separate absorption maxima (563 nm (2.20 eV) for HR, 532 nm (2.33 eV) for HG and 414 nm (2.99 eV) for HB) (Dartnall et al., 1983; Oprian et al., 1991) exhibited by the three cone visual pigments are all carried out by the chemically identical retinal. In other words, the absorption wavelengths of retinals change depending on the environment of the proteins (opsins) surrounding the retinals, and this phenomenon is called color tuning (Kochendoerfer et al., 1999). In the case of bovine rhodopsin, the crystal structures were solved (Palczewski et al., 2000; Okada et al., 2002) and many theoretical studies on the color tuning mechanism have been done by Olivucci *et al.* (Ferré and Olivucci, 2003; Andruniów et al., 2004; Coto et al., 2006), Buss *et al.* (Hufen et al., 2004; Sekharan et al., 2007), Elstner *et al.* (Wanko et al., 2005; Wanko et al., 2008), Morokuma *et al.* (Altun et al., 2008a; Altun et al., 2008b), and others (Gascón et al., 2006; Tomasello et al., 2009). In contrast, little attention has been paid to the study of cone visual pigments due to their unknown three-dimensional protein structures. At the time of our study, the excited state calculations for the cone visual pigments were done only by Trabanino et al. (2006) *.* However, this study was performed at the semi-quantitative CI-Singles level, and it was difficult to analyze the color tuning mechanism. In this study, we thus used the SAC-CI method, which is a quantitative electronic structure theory, to clarify the color tuning mechanism of the human cone visual pigments.

Since the crystal structures of the human cone visual pigments have not been available, we employed homology modeling structures [PDB ID: 1KPX, 1KPW, 1KPN for HR, HG, and HB, respectively (Stenkamp et al., 2002)], three-dimensional protein structures constructed by using the similarity of amino acid sequence with experimentally solved protein structures. The protonation states of charged amino acids were then evaluated for the homology modeling structure by Poisson-Boltzmann calculations using the MEAD program (Bashford and Gerwert, 1992) and the overall protein structures were further refined by the QM/MM geometry optimization. Thus, the protein structures of the three cone visual pigments were created.

Excited state calculations using the SAC-CI method were performed for these structures. As a result, the experimental values of the absorption energies were successfully reproduced with an RMS error of 0.05 eV (HB; 2.94, HG; 2.32, HR; 2.08 eV) (Fujimoto et al., 2008).

**FIGURE 1:**
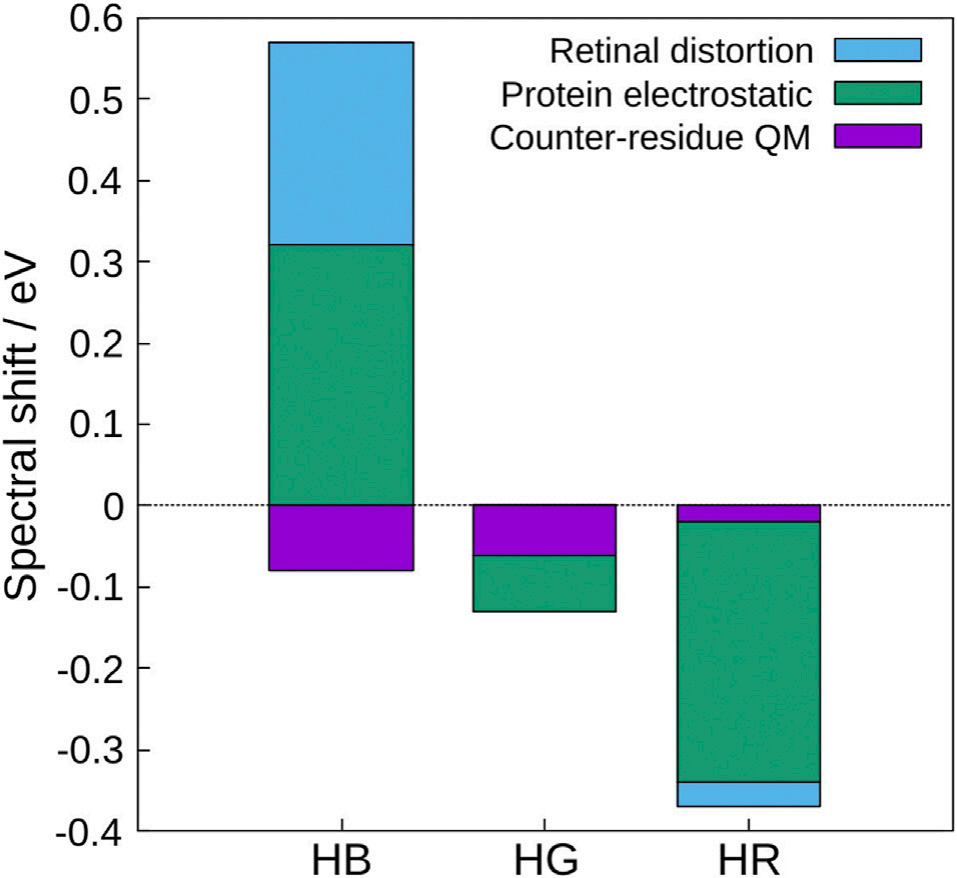
Contributions to the spectral shifts of HB, HG, and HR calculated by the decomposition of the shifted energy into the distortion effect of the retinal structure, the electrostatic effect created by the protein environment, and the quantum effect of the counter-amino acid (glutamate anion). The value of bovine rhodopsin was used as a reference.

Based on these results, the physical origin of the color tuning mechanism was explored (Fujimoto et al., 2008). To this end, the absorption energy for each of the three cone pigments was decomposed into three contributions: the distortion effect of the retinal structure, the electrostatic effect created by the protein environment, and the quantum effect of the counter-amino acid (glutamate anion). The quantum effect here refers to higher-order electronic interactions such as polarization and charge transfer (CT) caused by treating the counter amino acid as the QM atoms. The results show that the protein electrostatic effect makes the largest contribution to the absorption energy of the three cone visual pigments (green in Figure 1), and the distortion effect of the retinal structure also makes a large contribution in HB (light blue in Figure 1), but is not the main cause of the color tuning mechanism. Although the quantum effect of counter-amino acids was important in reproducing the absolute value of the absorption energy, there was no significant difference in the contribution of color tuning in the three cone pigments. From these results, only the electrostatic effect of opsin is discussed in this review.

**FIGURE 2:**
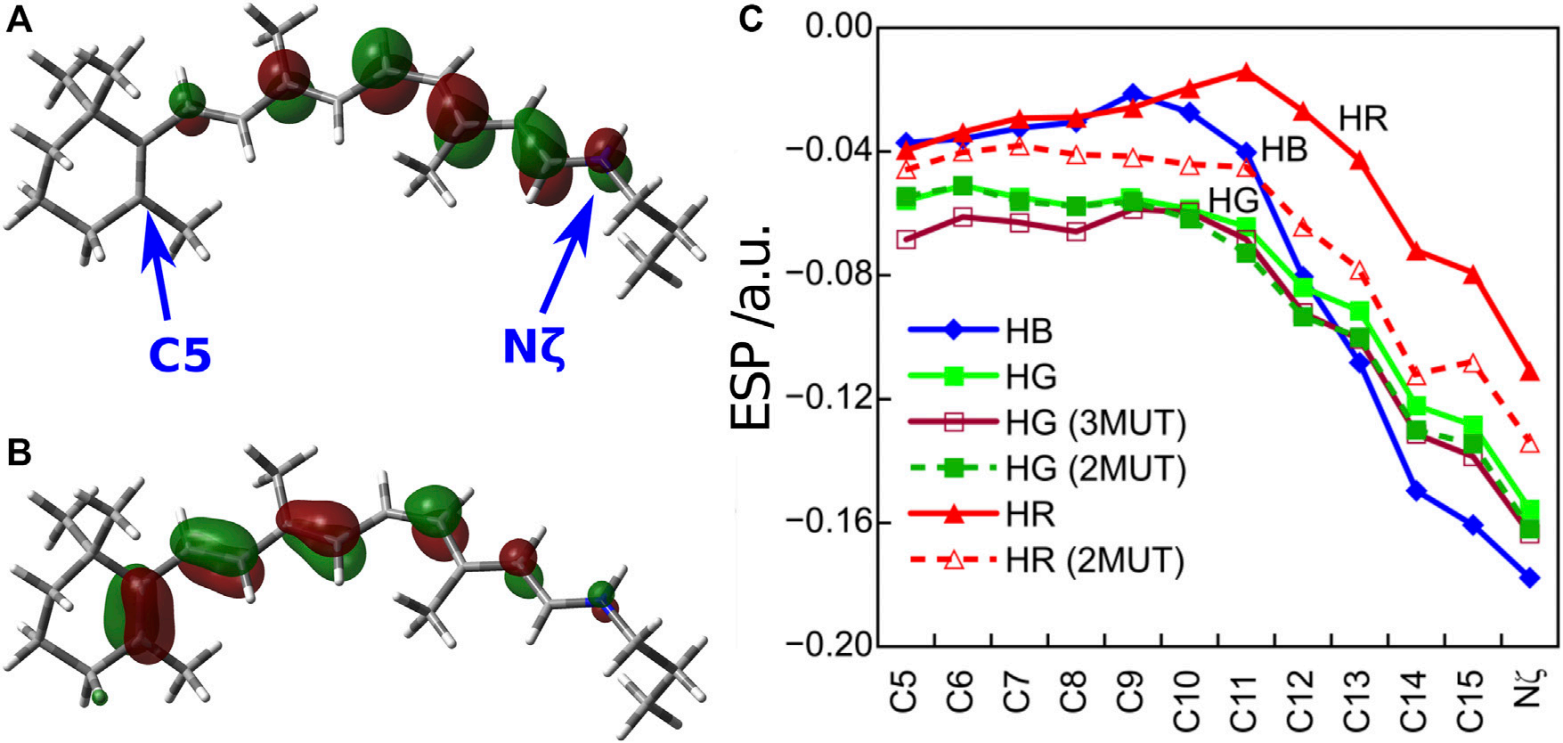
**(A)** LUMO and **(B)** HOMO distributions of the retinal chromophore. **(C)** ESP created by opsin on the retinal *π*-chain. Atoms C5 and Nζ are located on the β-ionone ring and Schiff base region, respectively.

In order to understand the protein electrostatic effect (Warshel and Levitt, 1976), let us consider 1) the character of the first excited state of the retinal chromophore and 2) the electrostatic potential (ESP) created by the protein environment of the cone visual pigments (Fujimoto et al., 2007). 1) The SAC-CI wavefunction showed that the first excited state of the retinal chromophore is characterized as a one-electron transition from the highest occupied molecular orbital (HOMO) to the lowest unoccupied molecular orbital (LUMO) (HOMO-LUMO transition). As can be seen from the HOMO and LUMO shown in Figures 2A,B, the HOMO is distributed on the left side (β-ionone ring side) and the LUMO on the right side (Schiff base side) of the retinal chromophore. Such an electronic transition between orbitals with different distributions implies that the first excited state of the retinal is of intramolecular CT character, which has also been confirmed by CASPT2 (Coto et al., 2006), SORCI + Q (Altun et al., 2008a), and experiments (Mathies and Stryer, 1976; Schenkl et al., 2005). Keeping in mind the character of the retinal chromophore, we next consider 2) the ESP by the protein environment. Note that this ESP is created by opsin on the retinal, not by the retinal itself. As shown in Figure 2C, the ESP created by opsin on the retinal *π*-chain is significantly negative in the region where LUMO is distributed (i.e., the Schiff base side). How does such a large negative ESP on the Schiff base side affect the molecular orbitals of the retinal? It is a specific destabilization of the energy level of LUMO. Negative ESP destabilizes the orbital energies of both the HOMO and LUMO, but specifically large negative ESP on the Schiff base side destabilizes the LUMO to a greater extent than the HOMO. Therefore, the difference between the HOMO and LUMO orbital energies of the retinal (HOMO-LUMO gap) is larger in the presence of the protein environment than in the gas phase (Fujimoto et al., 2007). This is the mechanism by which the protein electrostatic effect increases the absorption energy of the retinal chromophore.

How does the difference in the absorption energy of the three cone pigments arise? It comes from the difference in the degree of LUMO destabilization caused by the ESP of the three types of opsins (Fujimoto et al., 2009). As shown in Figure 2C, the degree of negative ESP on the Schiff base side relative to the β-ionone ring side increases in the order HR < HG < HB. As a result, the destabilization of the LUMO level is largest for HB and smallest for HR. Therefore, the HOMO-LUMO gap is the largest in HB and the smallest in HR, which leads to the difference in the absorption energy of the three cone pigments. From these results, we could clearly understand the color tuning mechanism of the human cone visual pigments caused by the different electrostatic effects (ESP) of opsin.

**FIGURE 3:**
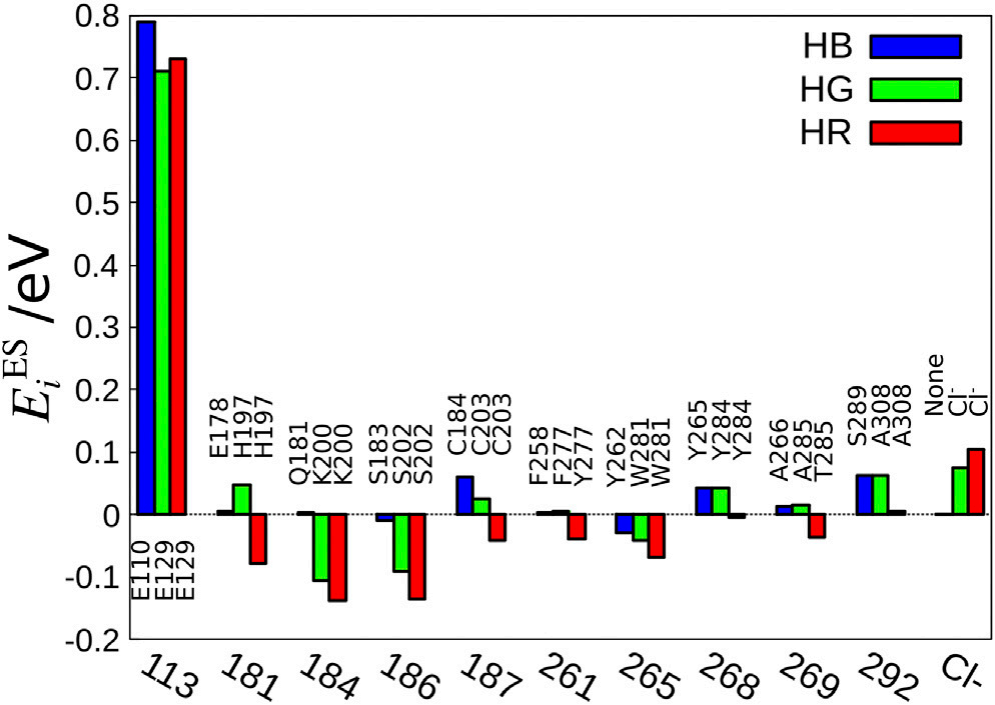
Amino acid sequences important for the color tuning. The amino acid number of bovine rhodopsin is shown as the standard value because the amino acid numbers are different among HB, HG, and HR.

The next question is, which amino acid makes the difference in the electrostatic effect among the three cone pigments? To answer this question, we have defined the electrostatic energy of each amino acid that contributes to the absorption energy.

$$E_{i}^{\text{ES}}=\sum_{a\in i}\int dr\frac{\Delta \rho \left(r\right)Q\left(r_{a}\right)}{\left|r-r_{a}\right|},$$ (1)

where Δρ(r) denotes the electron-density difference of retinal upon transition at position r and $Q\left(r_{a}\right)$ is the atomic charge of atom *a* of the amino acid *i*. Using this equation, we analyzed the amino acids with significant contribution to the absorption energy. As a result, we found that 10 amino acids specific to the amino acid sequences of the cone visual pigments and Cl^−^ ions contribute significantly to the electrostatic effect (Figure 3) (Fujimoto et al., 2009). Further analysis revealed that amino acids with OH groups are particularly important for the electrostatic effect (Fujimoto et al., 2009). The orientation of the OH group toward the retinal was found to be the main cause of the difference in the electrostatic effect in each cone pigment. The effect of dipole orientation of polar residues on color tuning has also been thoroughly investigated at the SORCI + Q level by Shtyrov et al. (2021) and their results were in agreement with ours. The electrostatic effect of charged residues depends on the position of the residue, and this effect has been discussed quantitatively by other groups (Ferré and Olivucci, 2003; Shtyrov et al., 2021).

## Excitation Energy Transfer in Xanthorhodopsin

**FIGURE 4:**
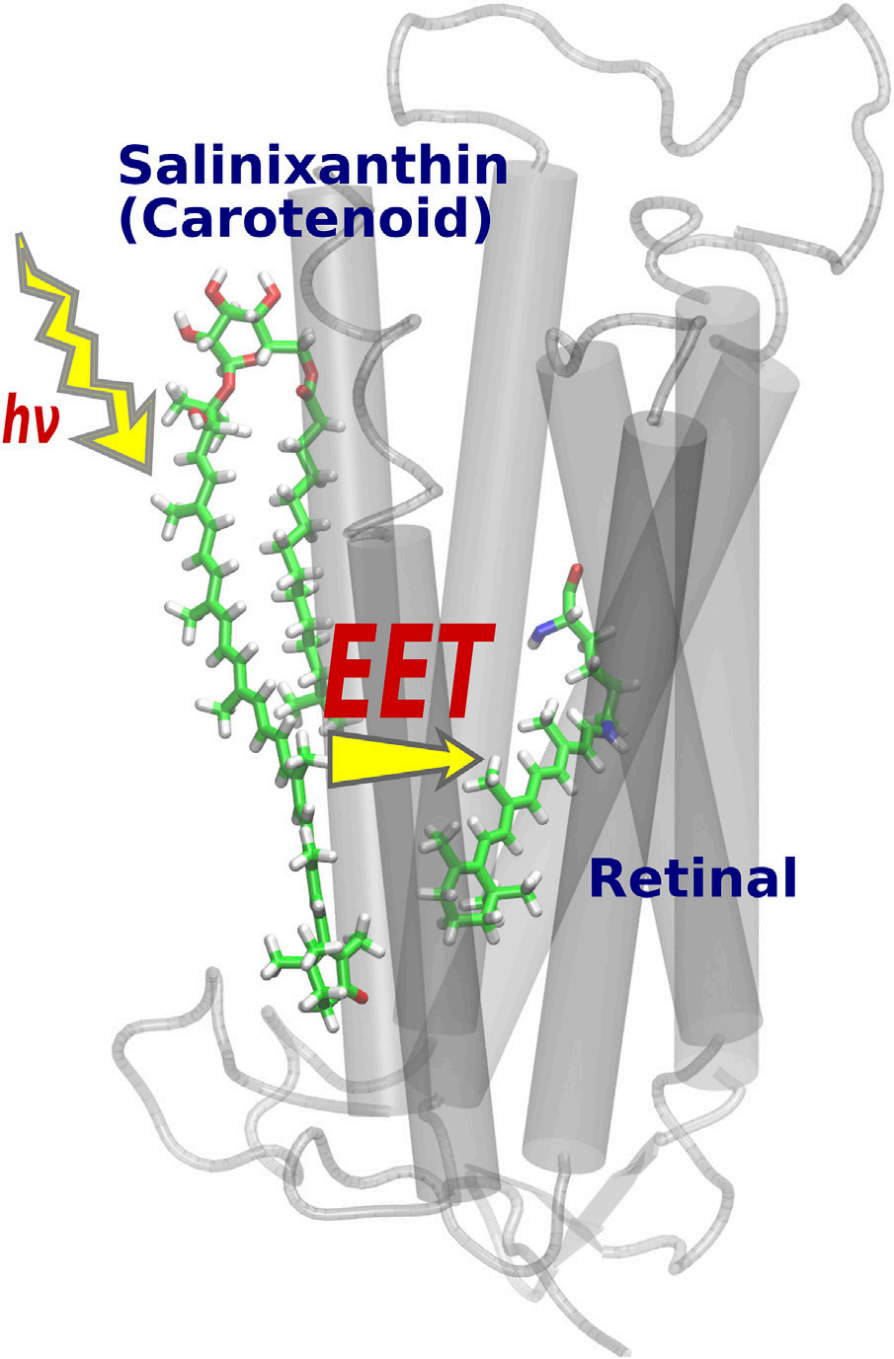
Optimized structure of XR. After light absorption by the salinixanthin added on the protein surface, the retinal is excited via EET.

EET is a phenomenon in which the electronic excitation of an acceptor molecule occurs simultaneously with the electronic deexcitation of a donor molecule. Fluorescence resonance energy transfer (FRET) (Berezin and Achilefu, 2010) is also a type of EET that occurs between fluorescent chemical compounds, and is widely used as an effective tool for dynamically visualizing the biological functions of gene products in living cells (Jares-Erijman and Jovin, 2003; Miyawaki, 2003). While it was widely known that the light-harvesting antenna of photosynthesis efficiently collects solar energy by utilizing EET phenomena *in vivo* (Scholes et al., 2011; Hu et al., 1997; Adolphs et al., 2010), Balashov *et al.* discovered a new EET system in XR, a member of retinal protein with proton pumping function (Lanyi and Balashov, 2008; Balashov et al., 2005). Here, salinixanthin (donor), a type of carotenoid bound to the surface of the XR protein, plays the role of a light-harvesting antenna, and the light energy captured there triggers electronic excitation of retinal (acceptors) *via* EET (Figure 4). The light-harvesting antenna in photosynthesis is formed by the aggregation of many chromophores [e.g., 27 bacteriochlorophylls in the case of light-harvesting complex 2 (LH2) (Papiz et al., 2003; Cogdell et al., 2006)], whereas in XR, only one molecule of carotenoid plays the antenna function (Luecke et al., 2008). Such a simple antenna structure in XR is theoretically tractable and useful for studying EETs in biomolecular systems.

In the theoretical study of XR, we need to pay attention to the small distance between the salinixanthin and retinal. The distance between the centers of mass of both molecules (13 Å) is smaller than the size of the molecules (∼36 Å) (Luecke et al., 2008), so the DD method cannot be applied to XR. In fact, the electronic coupling calculation using the DD method yields a result of −751 cm^−1^ (Fujimoto and Balashov, 2017), confirming that the experimental value of 160–210 cm^−1^ (absolute value) (Polívka et al., 2009) cannot be reproduced. Note that the sign of the electronic coupling cannot be determined because the experimental electronic coupling was derived from the squared form contained in Förster’s EET rate (Förster, 1948).

**FIGURE 5:**
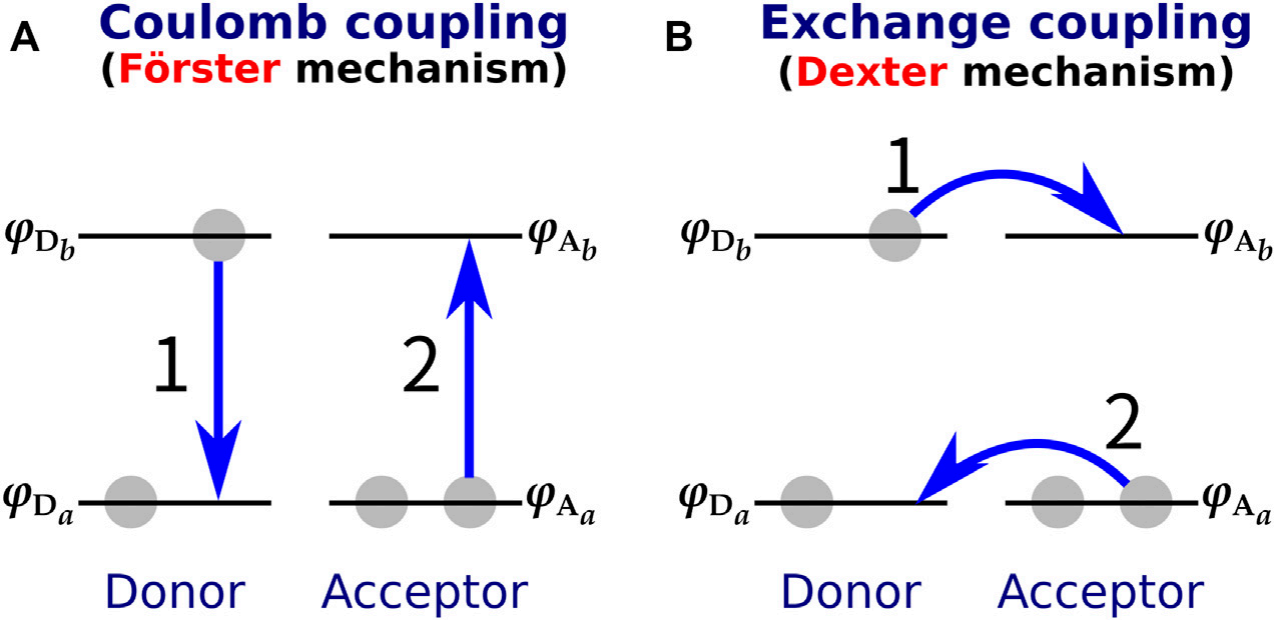
**(A)** Förster-type and **(B)** Dexter-type EET mechanisms. In the Förster type, EET is triggered by Coulomb coupling between donor and acceptor, while in the Dexter type it is caused by exchange coupling. The numbers 1 and 2 in the figure indicate the order of the electronic transitions.

With respect to the small donor-acceptor distance of XR, another consideration must be made in addition to the limited applicability of the DD method. The Förster-type (Förster, 1948) and Dexter-type (Dexter, 1953) theories are often used to explain the molecular mechanism of EET (Parson, 2007) (Figure 5). The Förster mechanism, which originates from the Coulomb interaction, is dominant when the intermolecular distance between donor and acceptor is large, while the Dexter mechanism, which is caused by the exchange interaction, dominates when the donor-acceptor intermolecular distance is small (Speiser, 1996; Berezin and Achilefu, 2010). Therefore, it has been considered that a Dexter-type EET may occur in XR with a small donor-acceptor distance (the smallest distance is 3.9 Å). However, it should be noted that most of the proposed methods for calculating the electronic coupling, such as the DD and TDC methods, can only describe the Coulomb interaction and cannot evaluate the exchange interaction. Therefore, it remains unclear whether the Dexter mechanism is involved in the EET of XR.

The TDFI method has the advantage of being able to calculate the exchange interaction $V_{\text{Exch}}$ in addition to the Coulomb interaction $V_{\text{Coul}}$ (Fujimoto, 2012; Fujimoto and Kitamura, 2013).

$$\begin{array}{c} V_{\text{EET}}=\int dr_{1}\int dr_{1}^{\prime }\frac{\rho_{I}^{\text{t∗}}\left(r_{1},r_{1}\right)\rho_{J}^{\text{t}}\left(r_{1}^{\prime },r_{1}^{\prime }\right)}{\left|r_{1}-r_{1}^{\prime }\right|}\;-\;\frac{1}{2}\int dr_{1}\int dr_{1}^{\prime }\frac{\rho_{I}^{\text{t∗}}\left(r_{1},r_{1}^{\prime }\right)\rho_{J}^{\text{t}}\left(r_{1}^{\prime },r_{1}\right)}{\left|r_{1}-r_{1}^{\prime }\right|}\\ \equiv V_{\text{Coul}}+V_{\text{Exch}}, \end{array}$$ (2)

where $\rho_{I}^{\text{t}}\left(r_{1},r_{1}\right)$ is a one-electron transition density of molecule *I*. In this study, the transition densities of retinal and salinixanthin were determined by SAC-CI method and time-dependent density functional theory (TD-DFT) (Runge and Gross, 1984) with the B3LYP functional (Lee et al., 1988), respectively. Using the TDFI method, the electronic coupling between the salinixanthin and retinal was calculated to be −227 cm^−1^, which includes −228 cm^−1^ for the Coulomb interaction and 1 cm^−1^ for the exchange interaction (Fujimoto and Balashov, 2017). These results show that the electronic coupling calculated with the TDFI method accurately reproduces the experimental values, and moreover, the EET between the carotenoid and retinal in XR is due to the Förster mechanism rather than the Dexter mechanism (Figure 5).

**FIGURE 6:**
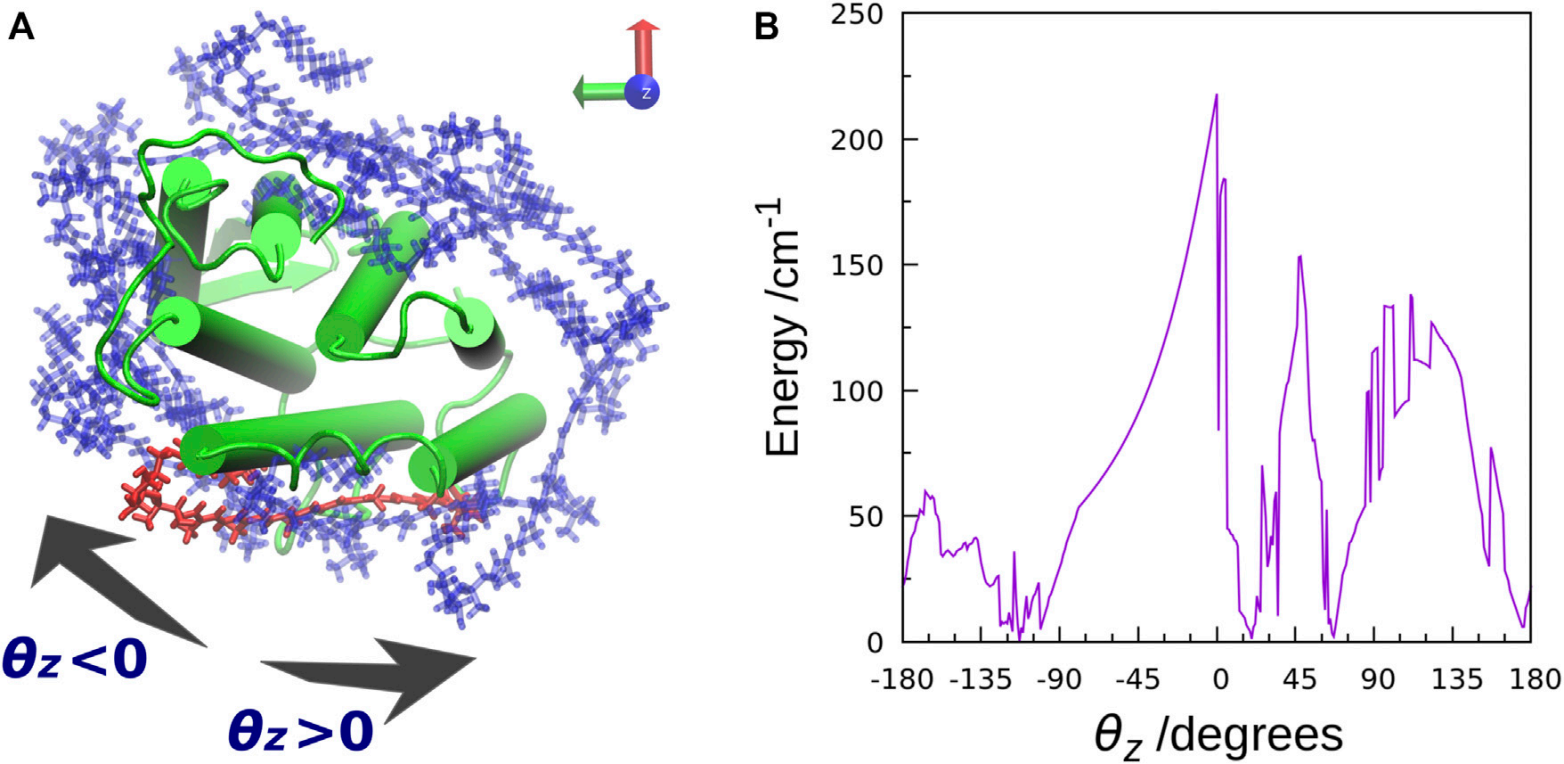
**(A)** Top view of the salinixanthin orientations generated around the XR protein using Euler angles. The rotation angle is taken as *θ*_*z*_. The native salinixanthin structure is shown in red, which is set to *θ*_*z*_ = 0. **(B)** Calculated electronic coupling energy between salinixanthin and retinal as a function of *θ*_*z*_.

The TDFI analysis of the electronic couplings provided us with a better understanding of the EET mechanism in XR. We also examined the effect of the orientation of the salinixanthin toward the retinal on the magnitude of the electronic coupling. First, we used Euler angles to generate artificial orientations of salinixanthin around the XR protein. Here, the salinixanthin was rotated in three dimensions on the surface of the XR protein, and 360 optimal conformations were generated (Figure 6A). The electronic couplings were then calculated for these structures (Figure 6B). The results show that the native carotenoid orientation (*θ*_*z*_ = 0°) yields the largest electronic coupling (EET efficiency: 40%). The second largest electronic coupling is 153.3 cm^−1^ at 48° (EET efficiency: 25%), which is 63 cm^−1^ smaller than the native orientation (0°). These results indicate that the native salinixanthin in XR is oriented to give large electronic coupling (high EET efficiency). The results in Figure 6B also show that the values of electronic coupling strongly depend on the salinixanthin orientation. An orientation with small electronic coupling corresponds to the fact that the light energy absorbed by salinixanthin is not transferred to the retinal. Therefore, this result indicates that the presence of salinixanthin on the XR surface does not necessarily lead to EET.

Archaerhodopsin 2 (AR2) is a member of retinal protein that also contains a carotenoid, bacterioruberin, but is known to have no EET function (Yoshimura and Kouyama, 2008). In order to investigate the difference in EET function between XR and AR2, a carotenoid, salinixanthin, mimicking the bacterioruberin orientation in AR2 was placed in XR, and electronic coupling calculations were performed for the structure. As a result, a very small value of 5.9 cm^−1^ was obtained (EET efficiency: 0.05%). This result demonstrates that we were able to reproduce the AR2-like state without EET function using XR by simply changing the salinixanthin orientation to the bacterioruberin one in AR2 without using bacterioruberin. At this stage, we cannot answer the question, “What is the role of bacterioruberin in AR2?”. However, we have found that at least the carotenoid orientation plays a major role in the expression of EET function.

## Effect of Electronic Coupling on Circular Dichroism Spectra

### Circular Dichroism Spectrum of Xanthorhodopsin

Electronic coupling (also called excitonic coupling) between molecules of the same species is known to induce two bands of opposite sign (biphasic bands) in CD spectra, and this phenomenon is called exciton-coupled CD (ECCD) (Harada and Nakanishi, 1983; Berova et al., 2000). ECCD bands also appear in biomolecules, and a typical example is the negative and positive CD bands observed in the bacteriorhodopsin (BR) trimer (Cassim, 1992; Pescitelli and Woody, 2012). However, there are few reports of ECCD in other biomolecules (Tsukamoto et al., 2013; Pescitelli et al., 2014; Iizuka et al., 2019), and the details of the mechanism are not well understood.

In general, the CD spectra of monomeric retinal proteins in the resting state show a single positive CD band at the absorption wavelength of the retinal (Cassim, 1992). On the other hand, the CD spectrum of XR is known to show negative and positive biphasic bands (Balashov et al., 2006). Three possibilities have been considered for the origin of the CD bands of XR: first, the distortion effect of the salinixanthin structure; second, the effect of electronic coupling between the salinixanthin and retinal; and third, the effect of multimer formation of XR. Here, the third effect refers to the retinal-retinal or salinixanthin-salinixanthin electronic coupling caused by the multimeric formation of XR (Smolensky Koganov et al., 2015). However, it is not clear what kind of multimeric structure is formed by XR. Therefore, we examined two of the above three possibilities, excluding the effect of multimer formation of XR.

In order to investigate the distortion effect of the salinixanthin structure (rotation of the C_6_-C_7_ single bond) on the CD spectrum, we calculated the CD spectrum by gradually changing the rotation angle of the C_6_-C_7_ single bond. As a result, it was confirmed that the sign of the CD band did not change at any rotation angle (Fujimoto and Balashov, 2017). Therefore, the C_6_-C_7_ single bond rotation of the salinixanthin structure is not a factor that characterizes the shape of the CD spectrum of XR.

**FIGURE 7:**
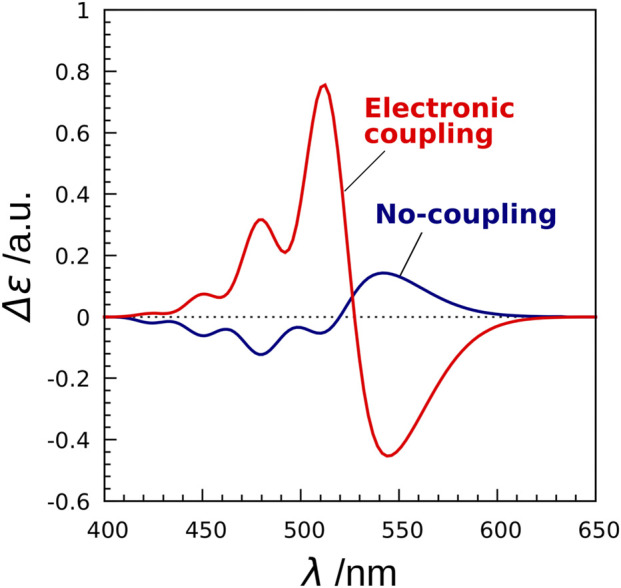
CD spectra of XR calculated by the exciton model and the no-coupling model. The no-coupling model does not take into account the electronic couplings in the exciton Hamiltonian.

Next, we investigated the effect of electronic coupling on the CD spectra (Fujimoto and Balashov, 2017). To this end, we employed two models (exciton model and no-coupling model) for the CD spectrum calculations. The Hamiltonian matrix of the exciton model is represented by

$$H=\left(\begin{array}{cc} E_{I} & V_{IJ}\\ V_{JI} & E_{J} \end{array}\right),$$ (3)

where the diagonal element $E_{I}$ denotes the excitation energy of molecule *I*, and the off-diagonal element $V_{IJ}$ is the electronic coupling between molecule *I* and *J*. The difference in the two models is the treatment of electronic coupling. In the exciton model, the value of salinixanthin-retinal electronic coupling calculated by the TDFI method was incorporated into the Hamiltonian matrix, while the value of electronic coupling was set to zero in the no-coupling model. The CD spectra calculated with the two models are illustrated in Figure 7. The spectrum obtained with the no coupling model showed two CD bands, but the spectral shape was opposite in sign to the experimental one. In contrast, the calculation by the exciton model showed two CD bands with the same sign as the experimental data. This means that the simple convolution of the CD spectra for the retinal and salinixanthin structures isolated from the protein structures cannot reproduce the experimental data. However, by considering the electronic coupling, the signs of the CD bands are reversed, which is consistent with the experimental CD shape. From these results, we conclude that the negative and positive CD bands observed in XR are formed by the effect of salinixanthin-retinal electronic coupling.

### Circular Dichroism Spectrum of Krokinobacter Eikastus Rhodopsin 2

In the case of XR above, we could not analyze the effect of multimer formation on the CD spectrum because there was no structural information on the multimer. To investigate the effect of multimer formation, we attempted to calculate the CD spectra of retinal proteins whose multimeric structures have been solved experimentally. Here, we focused on KR2, a light-driven sodium pumping retinal protein, which is closely related to XR in the phylogenetic tree (Shibata et al., 2018). Recently, KR2 has been attracting attention as an optogenetics tool in the field of neuroscience (Deisseroth et al., 2006; Wu et al., 2009; Kato et al., 2015). As mentioned above, the CD spectrum of monomeric retinal proteins in the resting state generally shows a single positive CD band originating from the photoexcitation of the retinal. In contrast, BR is known to produce negative and positive ECCD bands by forming a trimer (Cassim, 1992; Pescitelli and Woody, 2012). Therefore, it has been expected that KR2, which forms a pentamer (Gushchin et al., 2015), also exhibits biphasic CD bands similar to those of the BR timer. However, the measured CD spectrum of KR2 showed only a single positive CD band (Shibata et al., 2018). This result gave the possibility that the electronic coupling in the pentamer of KR2 does not affect the shape of the CD spectrum. On the other hand, the peak position of this CD band was red-shifted compared to the peak position of the absorption spectrum, suggesting that multimeric interactions may have affected the CD spectrum. These facts raised the question of whether the retinal-retinal electronic coupling in the KR2 pentamer affects its CD spectrum. However, since no theoretical studies on the CD spectra of KR2 pentamers have been performed, this molecular mechanism remains unclear.

**FIGURE 8:**
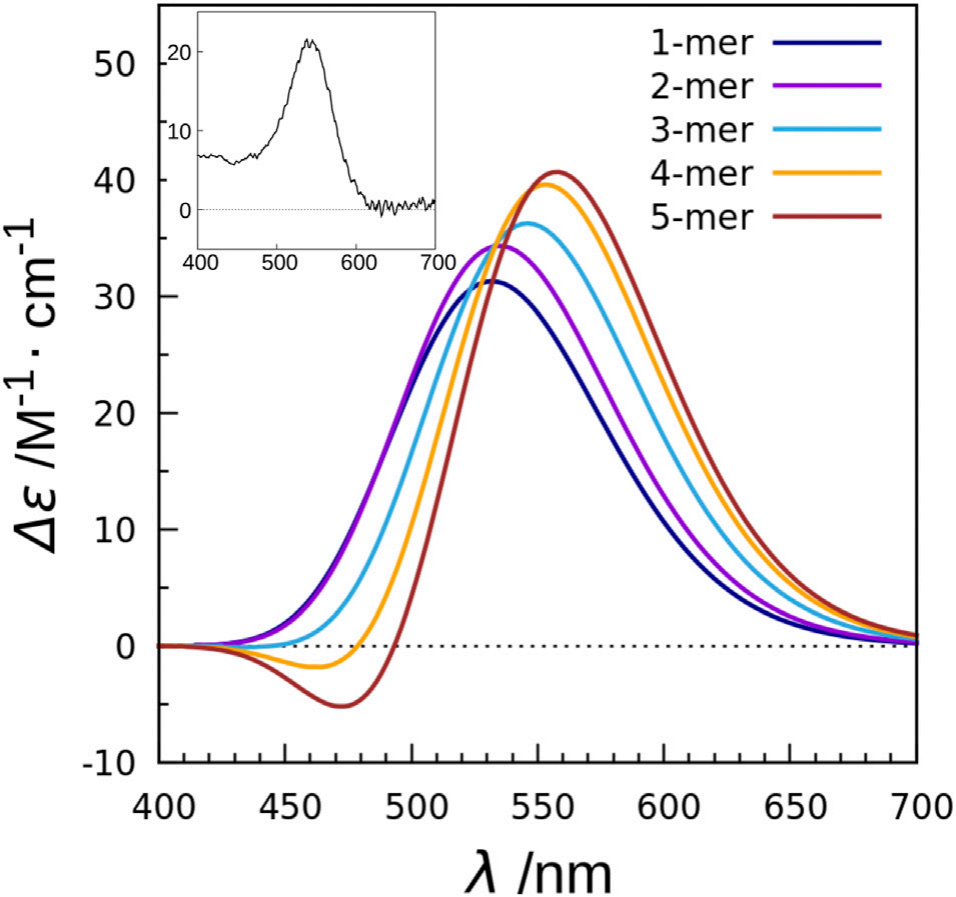
CD spectra calculated with the exciton model for monomer to pentamer in KR2. The values are given per monomer. The experimental absorption spectrum of KR2 (Shibata et al., 2018) is shown in the inset.

In order to clarify the mechanism, we performed calculations using a combination of the TDFI method and exciton model as in the case of XR described above, and analyzed the obtained spectra (Fujimoto and Inoue, 2020). This study also employed the SAC-CI method to determine the excitation energy and transition density of retinal. First, we calculated the electronic coupling between the retinals in the KR2 pentamer and obtained a value of 25 cm^−1^. This value is much smaller than the carotenoid-retinal electronic coupling in XR (227 cm^−1^). The TDFI analysis then revealed that the Coulomb interaction (25 cm^−1^) was the main cause of the electronic coupling, with no contribution from the exchange interaction (0 cm^−1^). This is due to the fact that the intermolecular distance between the retinals is 25 Å. Using the obtained electronic coupling values, the absorption spectra of the monomeric and pentameric structures of KR2 were calculated, and both showed almost the same absorption maxima. Next, the CD spectra were also calculated (Figure 8). The CD spectrum of the monomer showed a band at the same wavelength as the absorption spectrum. On the other hand, the CD spectrum of the pentamer showed an overall positive band, although there was a small negative peak at 471 nm. This positive CD band was red-shifted by 26 nm (0.11 eV) compared to the peak in the absorption spectrum, reproducing the red-shift observed in the experimental CD spectrum. In order to further analyze the effect of multimer formation, we additionally calculated the absorption and CD spectra using the no coupling model. As a result, the calculated CD spectrum showed a peak position at the same wavelength as the absorption spectrum. From these results, we found that the CD spectrum is affected by the retinal-retinal interaction due to multimer formation (Fujimoto and Inoue, 2020).

**FIGURE 9:**
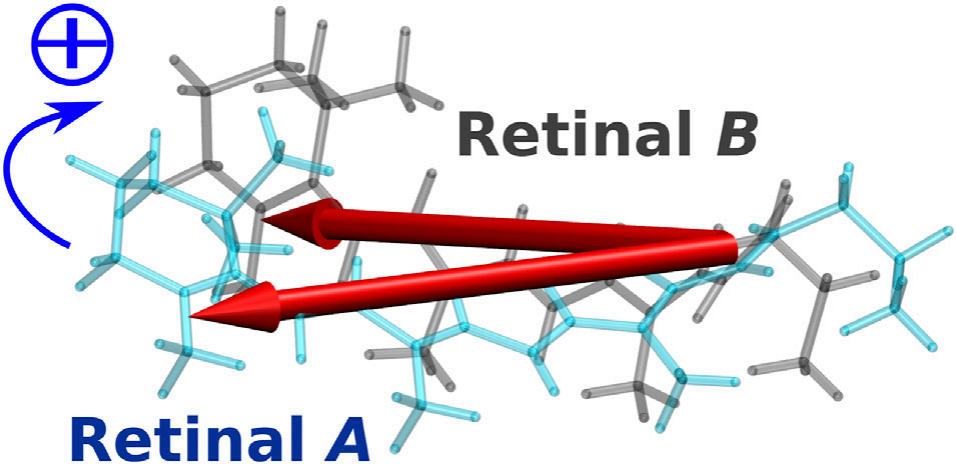
Intermolecular conformation of the two retinal chromophores (retinal *A* and retinal *B*) in KR2. The two red arrows represent the transition dipole moments of the two retinal chromophores.

In the case of the BR trimer, the effect of electronic coupling between the retinals generates negative and positive biphasic CD bands, with the negative band occurring on the longer wavelength side, which is called negative chirality. On the other hand, in the CD spectrum of the KR2 pentamer, a large positive band is generated on the longer wavelength side than a small negative band due to the effect of electronic coupling, which is regarded as positive chirality. This mechanism could be understood from the orientation of the retinals in the pentamer structure of KR2. As illustrated in Figure 9, the orientation of the transition dipoles of the two retinals shows a clockwise rotation from retinal *A* in the foreground to retinal *B* in the background (Fujimoto and Inoue, 2020). This clockwise rotation corresponds to positive chirality according to the exciton chirality method (Harada and Nakanishi, 1969; Harada and Nakanishi, 1983) by Harada and Nakanishi. From these results, we conclude that the positive band contributes significantly to the CD spectrum of KR2 due to the orientation between the retinals corresponding to the clockwise rotation of the two transition dipoles.

## Artificial Design of Light Absorption by Retinal Proteins

Several studies described above have shown that the optical properties of retinal proteins with respect to light absorption are strongly influenced by electrostatic interactions with surrounding proteins and by electronic couplings between chromophores. Here, we discuss the possibility of designing new optical properties by artificially altering these two interactions.

The first is the artificial control of the electrostatic interaction between the retinal and the surrounding protein. As mentioned above, the first excited state of the retinal chromophore has the character of intramolecular CT, so by changing the magnitude of the ESP formed by the protein along the retinal *π*-chain (Figure 2C), the absorption wavelength of the retinal can be changed. A direct way to change the ESP is to introduce charged amino acids virtually on a computer. Here, the ESP formed by the charged residues needs to be quantified according to the position of the residues (Ferré and Olivucci, 2003; Shtyrov et al., 2021). However, it should be noted that the three-dimensional structure of the mutant protein obtained by this operation may be quite different from the real one due to the change in the charge balance of the protein. The evaluation of the homology model of rhodopsin is discussed in Ref. (Nikolaev et al., 2018). In addition to charged amino acids, the introduction of amino acids with OH groups, such as serine and tyrosine, is an effective way to modify the ESP (Figure 2C) (Fujimoto et al., 2009). Mutant experiments by Asenjo et al. (1994) revealed that three OH amino acids in the vicinity of the β-ionone ring of retinal contribute significantly to the longer absorption wavelength of HR than that of HG. We have shown that the orientation of the OH group in these amino acids contributes to the red shift, and that the orientation of the oxygen of the OH group toward the β-ionone ring causes a specific negative ESP on the β-ionone ring side, which results in a red shift in the absorption wavelength of HR (Fujimoto et al., 2009). The contribution of the OH group orientation to the red shift was also confirmed by the theoretical mutants, in which the neutral amino acids Ala180, Phe277, and Ala285 in HG were replaced by the corresponding OH group amino acids Ser, Tyr, and Thr in HR, respectively (Fujimoto et al., 2009). The absorption energies of the three mutant structures were calculated to be 2.20 eV, which is in good agreement with the experimental value of 2.33 eV. Although the above example concerns amino acids that produce the difference in absorption wavelengths between HR and HG, we believe that this strategy can be applied to the artificial design of absorption wavelengths for a number of retinal proteins. We note here that accurate prediction of the three-dimensional structure of the mutant protein will be a critical process for the success of this strategy. Electrostatic effects on the color tuning of rhodopsin are also known to be due to the position of internal water molecules (Tsutsui and Shichida, 2010; Nikolaev et al., 2020), or indirectly due to the substitution of one residue causing rearrangement of others (Ryazantsev et al., 2012). Taking these effects into account when constructing three-dimensional structures will lead to more accurate artificial designs.

The second is to modulate the electronic coupling by adding some chromophore on the surface of retinal protein. As mentioned above, XR acquired a new optical absorption property with EET by adding the salinixanthin on the protein surface. Our analysis for XR revealed that controlling the carotenoid-retinal orientation is more important than the type of carotenoids to increase the EET efficiency (Fujimoto and Hayashi, 2009). This analysis was done by using Euler angles to exhaustively search for the salinixanthin conformations, but other approaches are also possible. The author is also working on computational drug discovery and has developed a method for predicting the binding pose of a ligand to a protein, called molecular docking. Accurate molecular docking methods, such as the artificial bee colony (ABC) (Karaboga and Basturk, 2007) algorithm-based docking method developed by the authors (Uehara et al., 2015), will enable the exploration of ligands to be added on the surface of retinal proteins, which is expected to lead to the construction of new EET systems. An attempt to add salinixanthin to retinal proteins other than XR have already been reported (Misra et al., 2019), but the use of computational methods prior to such challenges will further expand the scope of research.

## Future Perspective

In this review, we have presented the molecular mechanisms of retinal proteins (human cone visual pigments, XR, and KR2) revealed by using three types of quantum chemical calculations. For details of the methods and mechanisms, please refer to the original papers.

We have described the Förster-type and Dexter-type mechanisms of EET of XR, but the author has also studied another type of EET mechanism called “EET *via* CT states (Harcourt et al., 1994; May and Kühn, 2011)” and has shown that this third mechanism strongly contributes to ethylene dimer (Fujimoto, 2012; Fujimoto, 2015). In the future, we would like to explore the effects of this mechanism on biomolecular systems.

The CD spectral analysis of KR2 revealed that the retinal-retinal electronic coupling in the pentamer structure affects the shape of the CD spectrum. A comprehensive understanding of the relationship between protein multimer formation (Brown and Ernst, 2017) and CD spectral shape (Shibata et al., 2018) is a future challenge.

A technical challenge in dealing with protein multimers is the reduction of computational cost. In the electronic coupling calculations, the author has developed the transition charge, dipole, and quadrupole from ESP (TrESP-CDQ) method (Fujimoto, 2014), which is less computationally expensive than the TDFI method. The use of such a method is expected to contribute to efficient analysis for protein multimers.

The methods presented in this review do not consider the effects of protein fluctuations. To what extent do protein fluctuations affect the excited state properties (optical properties) of chromophores such as retinal and carotenoids? To investigate it, benchmark calculations for excited states have been made using a combination of semiempirical method and MD simulation (Bold et al., 2020). By adding statistical processing to the method presented in this study, we hope to quantitatively clarify the effect of protein fluctuation on the optical properties of rhodopsin, which will contribute to the elucidation of the molecular mechanism in more detail.
